# Physician Stress During Electronic Health Record Inbox Work: In Situ Measurement With Wearable Sensors

**DOI:** 10.2196/24014

**Published:** 2021-04-28

**Authors:** Fatema Akbar, Gloria Mark, Stephanie Prausnitz, E Margaret Warton, Jeffrey A East, Mark F Moeller, Mary E Reed, Tracy A Lieu

**Affiliations:** 1 Department of Informatics Donald Bren School of Information and Computer Sciences University of California, Irvine Irvine, CA United States; 2 Division of Research Kaiser Permanente Northern California Oakland, CA United States; 3 The Permanente Medical Group Oakland, CA United States; 4 Department of Adult and Family Medicine Kaiser Permanente Richmond, CA United States; 5 Department of Adult and Family Medicine Kaiser Permanente San Rafael, CA United States; 6 Department of Adult and Family Medicine Kaiser Permanente Napa, CA United States

**Keywords:** electronic health records, stress, wearables, HRV, inbox, EHR alerts, after-hours work, electronic mail, physician well-being, Inbasket

## Abstract

**Background:**

Increased work through electronic health record (EHR) messaging is frequently cited as a factor of physician burnout. However, studies to date have relied on anecdotal or self-reported measures, which limit the ability to match EHR use patterns with continuous stress patterns throughout the day.

**Objective:**

The aim of this study is to collect EHR use and physiologic stress data through unobtrusive means that provide objective and continuous measures, cluster distinct patterns of EHR inbox work, identify physicians’ daily physiologic stress patterns, and evaluate the association between EHR inbox work patterns and physician physiologic stress.

**Methods:**

Physicians were recruited from 5 medical centers. Participants (N=47) were given wrist-worn devices (Garmin Vivosmart 3) with heart rate sensors to wear for 7 days. The devices measured physiological stress throughout the day based on heart rate variability (HRV). Perceived stress was also measured with self-reports through experience sampling and a one-time survey. From the EHR system logs, the time attributed to different activities was quantified. By using a clustering algorithm, distinct inbox work patterns were identified and their associated stress measures were compared. The effects of EHR use on physician stress were examined using a generalized linear mixed effects model.

**Results:**

Physicians spent an average of 1.08 hours doing EHR inbox work out of an average total EHR time of 3.5 hours. Patient messages accounted for most of the inbox work time (mean 37%, SD 11%). A total of 3 patterns of inbox work emerged: inbox work mostly outside work hours, inbox work mostly during work hours, and inbox work extending after hours that were mostly contiguous to work hours. Across these 3 groups, physiologic stress patterns showed 3 periods in which stress increased: in the first hour of work, early in the afternoon, and in the evening. Physicians in group 1 had the longest average stress duration during work hours (80 out of 243 min of valid HRV data; *P*=.02), as measured by physiological sensors. Inbox work duration, the rate of EHR window switching (moving from one screen to another), the proportion of inbox work done outside of work hours, inbox work batching, and the day of the week were each independently associated with daily stress duration (marginal *R^2^*=15%). Individual-level random effects were significant and explained most of the variation in stress (conditional *R^2^*=98%).

**Conclusions:**

This study is among the first to demonstrate associations between electronic inbox work and physiological stress. We identified 3 potentially modifiable factors associated with stress: EHR window switching, inbox work duration, and inbox work outside work hours. Organizations seeking to reduce physician stress may consider system-based changes to reduce EHR window switching or inbox work duration or the incorporation of inbox management time into work hours.

## Introduction

### Background

Inbox management is an important component of electronic health record (EHR) work for physicians and a key potential stressor [1]. Through their EHR inbox, physicians receive messages from other physicians, staff, and patients. Studies of inbox management in other professions repeatedly report inbox management as a source of stress due to the time it takes to go through an ever-increasing volume of emails, the task demands associated with emails, and the interruptions they create [2-4]. Similarly, EHR inbox management has been identified as a possible contributor to physician stress and burnout [5,6]. To understand the relationship between EHR adoption and use and stress, it is critical to examine how physicians spend time on the EHR inbox.

Although several studies have addressed the stress or burden related to EHR use, there are two main limitations in previous work. First, scant research focusing on the inbox component of the EHR exists [1,5,7,8]. Second, previous studies relied on self-reported stress measured at a single time point (or a few time points) [5], which fails to capture the detailed and continual stress and EHR work patterns throughout the day and is prone to bias [9,10].

Our study investigates physicians’ EHR inbox use patterns and associated stress, as measured unobtrusively and continuously by EHR system logs and wearable sensors. The objectives of this study are as follows:

1. Collect EHR use and stress data through unobtrusive means that provide objective and continuous measures.
2. Cluster and visualize distinct EHR inbox work patterns and identify their characteristics.
3. Identify physicians’ daily stress patterns.
4. Evaluate the association between EHR inbox work characteristics and physician stress.

### Previous Work on Physician Workload Related to the EHR and EHR Inbox

Studies have noted the burden of EHR digital work for physicians [11-13]. EHR-related factors that could lead to physician stress and burnout include the extra time needed, often beyond work hours, to complete EHR-related work [14-17], usability issues [18-20], risks associated with errors [21], and taking time out from face-to-face interactions with patients [22].

For EHR inbox management, a 2017 study [14] using EHR logs found that time spent in the inbox accounted for 24% of total EHR time, and of the time spent in the inbox, a larger proportion was spent after work hours compared with the time spent on other EHR activities. A study reported that 86% of surveyed physicians worked outside of work hours to respond to inbox messages [23], whereas another study reported that 37% of inbox work was done outside of work hours [24]. In addition to the time it takes within and outside of work hours, inbox-related burden has been attributed to the volume and source of EHR messages [5,7] and information overload from notifications (ie, asynchronous alerts) [25]. A 2012 study based on EHR logs [26] found that primary care physicians (PCPs) received a mean of 56.4 alerts per day and spent an estimated average of 49 minutes per day processing their alerts. A more recent study [1] found that PCPs received a mean of 77 (SD 38) inbox message notifications per day compared with the 30 notifications for specialists. Message quantity has been associated with increased attention switching and inbox work duration [27]. However, although these studies quantified EHR inbox–related factors and measured self-reported workload, well-being, or burnout at a single time point, they did not measure daily stress associated with EHR inbox use.

### Unobtrusive Sensing of Stress

One of the main limitations of previous studies on EHR and stress is the reliance on self-reported measures of well-being and burnout collected at a single time point [7,18]. In addition to not directly measuring stress per se, self-report approaches have several limitations for stress monitoring in the workplace. When people subjectively report how they feel, their evaluation could be affected by memory bias and emotion recognition, regulation, and expression biases [9,10,28,29]. Administering surveys for self-reports can also be disruptive, as they require the full cognitive attention of the user and do not allow continuous or frequent measurement that could be correlated with inbox use.

Advances in wearable sensors and algorithms that filter and analyze their data enable objective, continuous unobtrusive sensing of physiological measures directly associated with stress, such as heart rate variability (HRV). HRV is the variation in time between one heartbeat and the next. When relaxing and recovering, HRV increases, and it decreases during stress [30-32]. Thus, measuring HRV throughout the day can provide an objective and continuous measure of stress and relaxation, which can be used to identify events associated with stress in more granularity than is possible with self-reports.

Compared with other physiological stress measures that can be obtained from wearable sensors in daily life, HRV is more reliable in real-world settings (outside the laboratory). For example, skin conductance (ie, electrodermal activity [EDA]) can be difficult to measure in dry, indoor air-conditioned settings as the electrodes rely on sweat to measure conductance. In addition, some people do not naturally produce adequate EDA signals [33]. HRV sensors in wrist-wearable devices are light based (photoplethysmography sensors) and are more commonly used in consumer-grade wearables.

HRV is affected by a number of factors other than stress, such as physical activity and overall health. Thus, HRV as a measure of stress is most reliable for healthy participants in sedentary settings. Previous studies used HRV from wearable devices as a measure of stress in office settings where participants were working on a computer [34-37], making this method applicable to computer-based work by physicians.

## Methods

### Study Setting

Data collection was conducted at one of the largest medical groups in the United States. The medical group has 9200 physicians and serves 4.4 million members in 21 hospital-based medical centers.

Since 2008, the participating medical group has been using a comprehensive EHR (Epic Systems) that integrates inpatient, emergency, and outpatient care, including primary care, specialty, laboratory, pharmacy, and imaging data. The EHR inbox, named the Inbasket, receives messages sent by patients via a portal website (also available through patient-facing mobile apps) and messages from other physicians, clinical staff, the pharmacy, laboratory, and other departments. Physicians can access the Inbasket on computers or mobile devices. Physicians are expected to respond to each patient message within 2 business days. Patients are encouraged to use the messaging functionality of the EHR to enhance access to their physicians and the care experience.

Typical work hours when clinical settings are open and patient appointments are booked are from 8:30 AM to 12:30 PM and 1:30 PM to 5:30 PM. Clinic time is dedicated to patient appointments, which are conducted in person in the clinic or via telephone or video telemedicine. Some physicians also do clinical work during weekends, with work hours that might differ from weekdays.

### Recruitment and Protocol

Adult PCPs from 5 medical facilities within the medical group were recruited. Between 7 and 12 physicians were enrolled at each facility, with a total of 47 eligible physicians enrolled.

Physicians were eligible if they performed outpatient clinical work for at least 3.5 days a week. Physicians who were taking cardiac medications, had pacemakers or defibrillators, or had been diagnosed with cardiac arrhythmias were not eligible because of the interference of these factors with the HRV-based stress measure. Eligibility was confirmed via a recruitment email.

After obtaining written informed consent, the staff assigned a wearable device with heart rate sensors (Garmin Vivosmart 3) and configured the associated mobile apps (Garmin Connect and Tesserae Phone Agent [38]) on the physician’s work-issued mobile phone. The apps streamed data from the wearable device via Bluetooth and uploaded the data to a server. The research team also installed an experience sampling app [39] on the physician’s mobile phone to send short questions at specified times (see the *Experience Sampling* section). At enrollment, physicians completed a brief 5-question written survey about their EHR inbox management and stress.

Physicians were asked to wear the device and respond to the daily short survey prompts for 7 consecutive days and keep their phones and the wearable device charged. Physicians were free to keep their wearable devices after data collection. The study protocol was approved by the institutional review board of Kaiser Permanente Northern California.

### Data

#### EHR System Logs

We used system access logs, which contained granular timestamped data on the Epic system EHR use. We created hourly time bins and variables from the log data to quantify how time was attributed to different activities and different types of inbox messages per hour. These variables, which were collected for every hour, included the number of minutes spent in the EHR, the number of minutes spent in the inbox, the number of minutes spent working on each inbox message type, the number of tasks performed, and the number of window switches (ie, clicking a new computer window).

We categorized the system-generated labels for message type description into high-level categories by analyzing the frequency of the labels along with input from our clinical collaborators who are familiar with the meanings and patterns of different types of messages. This approach resulted in 4 message types: (1) messages from patients; (2) results, such as laboratory test results; (3) requests, which ask the physician to perform an action such as approving a medication refill or signing clinical orders; and (4) informational and administrative messages. No message content or metadata (ie, sender, receiver, and message ID) were collected.

#### HRV-Based Measure of Stress

The device used to measure HRV (Garmin Vivosmart 3) was a wrist-worn device with an optical heart rate sensor. It produces a *stress score* based on HRV in still moments (ie, excluding times with physical activity that interfere with HRV readings) and accounts for the physiological norm of each user. The stress score ranges from 0 to 100 and is provided via the Garmin application programming interface as 3-minute averages of the real-time stress scores generated on the device. The stress analysis method used by the device has been empirically tested and validated [40]. Garmin heart rate sensors were also compared with other devices and were found to be among the most accurate devices [41-44].

In our analyses, the HRV-based stress measure was the duration (number of minutes) of medium and high stress (stress score of >50). We excluded low stress periods (scores from 25 to 50) because a certain amount of physiological stress indicates arousal which is expected (and needed) for performing daily tasks [45].

There were some gaps in the continuous HRV stress data (see the *Analysis* section). Missing HRV stress data could be attributed to loose fitting of the sensors on the wrist, removing the device for charging, or forgetting to wear the device or physical activity. We set a minimum of 20 minutes of HRV data per hour for hourly stress measures and 2 hours of data for daily measures to be included in the analyses. We further report the number of valid minutes of data on which each reported stress measure is based.

#### Experience Sampling

During the data collection period, physicians received 3 short daily surveys via the experience sampling app. The survey consisted of 3 questions asking physicians to rate their stress in the last 5 minutes (from no stress to high stress), their arousal level (from low energy to high energy), and their mood (from unpleasant to pleasant). The experience sampling app triggered a phone notification asking physicians to take the survey 3 times a day: morning (between 9:30 AM and 10:30 AM), lunchtime (between 1 PM and 1:30 PM), and afternoon (between 3 PM and 4 PM). The survey expired 45 minutes after the notification if not opened.

#### Self-Reported Inbox Management Strategies and Related Stress

At enrollment, physicians were asked to complete a 5-question survey on their strategies for and feelings about Inbasket (their EHR inbox) management. Physicians were asked to indicate how distressful they found inbox management and whether they had responsibilities that restricted their ability to work before or after formal work hours.

#### Physician Characteristics

We also obtained physicians’ age, sex, years of experience, and full-time equivalent (FTE) status, which is a measure of clinical workload where 40 hours per week of scheduled work is 1.0 FTE. According to internal analyses by the medical group, FTE is strongly correlated with the patient panel size for physicians.

### Analysis

We used the Gaussian Mixture Models clustering algorithm [46] to find distinct patterns of inbox work. Features in the model included the distribution of inbox time in work hours and outside of work hours contiguous and noncontiguous to work hours. Multiple feature and cluster counts were tested, and the clustering that yielded more balanced clusters and had a reasonable silhouette score (a score that indicates how distinct or overlapping the clusters are) [47] was selected.

To capture whether physicians dedicated certain blocks of time for inbox work or consistently checked their inbox throughout the day, we defined days with inbox work batching as days where 70% or more of the total inbox work duration occurred in 3 separate blocks of time or less. With consistent inbox checking, a uniform distribution of inbox duration over the day would typically be observed, whereas batching would show 2-3 daily peaks of high inbox duration [35]. We compared this measure across clusters and used it as an independent variable in the mixed effects model along with the other EHR inbox use characteristics.

To compare clusters (ie, groups of different inbox work patterns), each comparison variable was tested for normality and homogeneity of variances before conducting an analysis of variance for normal distributions with equal variances or the Kruskal-Wallis test otherwise. For pairwise comparisons, a posthoc analysis was conducted using the Tukey honestly significant difference test for normally distributed variables and Dunn test for nonparametric posthoc comparisons. Categorical variables were tested using the Chi-square test.

To plot hourly stress patterns, we removed hours with less than 20 minutes of valid HRV data to avoid overestimating the stress duration as a ratio of the measurement period (the measurement period being valid HRV measurement duration). From a total of 4245 hours, this filter removed 1177 hours (27.73%) of the workdays’ HRV data. For daily stress measures, workdays with less than 2 hours of valid HRV data were removed from the analysis, as well as workdays that are Saturdays or Sundays, and those with no inbox activity. This filter removed 21 days in total, keeping 178 workdays for the daily stress analyses (cluster comparison and a regression model).

We investigated the relationship between daily EHR inbox use and stress through a generalized mixed effects model with physicians as random effects. A Poisson distribution was used to represent stress minutes as events within the observation period (ie, valid HRV minutes as an offset in the model). The distribution of the dependent variable (ie, stress duration) was right skewed, as expected in a Poisson distribution. The independent variables were centered (ie, mean subtracted). The variance inflation factor was under 5 for all independent variables, indicating that multicollinearity was not a problem. Several models were compared, starting with a base model and incrementally adding variables, to ensure that the improvement in the model justified the added complexity of adding variables. The model with the lowest Akaike information criterion and highest marginal (fixed effects) *R^2^* is presented.

## Results

### Participants

The 47 physicians (32/47, 68% female) were aged an average of 43.83 years (SD 9.51; range 31-68), had an average of 15.17 (SD 9.93; range 4-42) years of experience in medicine, and had an average FTE of 81% (SD 14%). On average, physicians in the data set had 5.26 workdays (SD 0.94) and 2.74 nonworkdays (SD 0.94) over the 8 days of data collection (the day of enrollment plus 7 days in the study).

The HRV-based stress analyses included 42 physicians, because 5 physicians (1 male and 4 female) had technical issues, thereby causing loss of the wearable device data.

The inbox strategies and stress survey was completed by 44 physicians.

### Three Distinct Patterns of EHR Inbox Work

On workdays, physicians spent an average of 3.5 hours (SD 0.69) in the EHR, of which 1.08 hours (SD 0.38) were spent doing inbox work. On nonworkdays, physicians spent an average of 23.88 minutes (SD 36.3) in the EHR, including an average of 13.78 minutes in inbox (SD 23.78). The majority of time in the inbox was spent on patient messages (mean 37%, SD 11%), followed by laboratory results (mean 31%, SD 8%), requests (mean 20%, SD 6%), and administrative messages (mean 13%, SD 5%).

**Figure 1:**
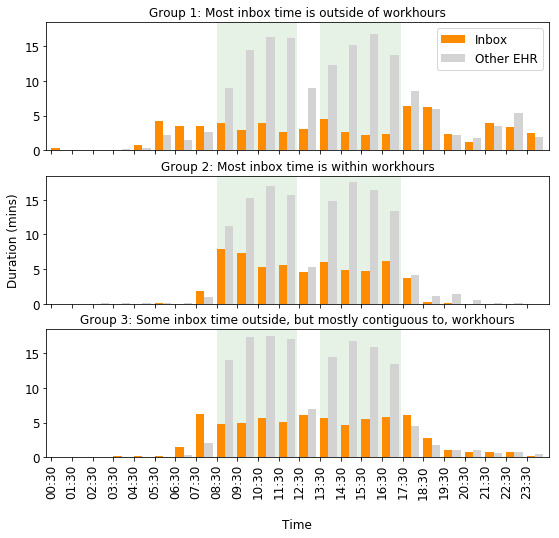
Temporal patterns of inbox and other EHR work. The green background indicates work hours. EHR: electronic health record.

Using the Gaussian Mixture Models clustering algorithm, we found 3 temporal patterns of work, with a silhouette score of 0.41, indicating moderate separation between these clusters (ie, distinct groupings). Figure 1 shows the average hourly time spent in the inbox and other EHR work (such as charting and order entry) for physicians in each cluster. Group 1 (n=10) represented physicians who spent time in the inbox outside work hours, in the evenings and early mornings; group 2 (n=17) represented physicians who worked mostly within work hours; and group 3 (n=20) represented physicians who spent some time on inbox work after hours that were mostly contiguous to work hours.

Free-text responses from the survey on inbox management strategies supported these computationally generated inbox work patterns. Responses from physicians in group 1 indicated working beyond work hours, either by staying late in the office or taking work home. Some representative comments were as follows. A physician in group 1 reported, “I find when I sacrifice sleep to do more at home, I’m too tired during the day and I’m very inefficient at night,” indicating that they were working late at night. Physicians in group 2 indicated working mostly within work hours. For example, one physician in this group asserted, “I arrive around 8:30 and prefer to leave around 5:30.” Another stated: "I just like to work and finish work during my allotted work time. I do not like to work at other times or at home."

Physicians in group 3 also indicated not taking work home but at the cost of staying late in the office to clear their inbox. For example, a physician in group 3 said, “I generally try not to take work home [...] so often stay very late to clean out inbasket.”

Physician characteristics (age, sex, years of experience, and FTE) did not show statistically significant differences across the 3 work patterns. In terms of EHR use, total daily time spent on inbox work and other EHR work on workdays (24-hour period) did not differ across groups (*P*=.38 and *P*=.15, respectively). However, as shown in Table 1, physicians in group 1 spent more time in the inbox after work hours compared with other groups, both in minutes and as a percentage of daily inbox time (*P*<.001). Posthoc comparisons showed that all the groups differed from each other. Group 1 also spent more time in the inbox work on nonworkdays (*P*=.03).

**Table 1:** Comparing inbox use characteristics across 3 work patterns.

Inbox use characteristics			Group 1, mean (SD)		Group 2, mean (SD)		Group 3, mean (SD)		*P* value
**Clustering factors (percentage of all-day inbox duration)**									
	Work hours inbox duration	37 (12)		82 (8)		62 (9)		<.001	
	Outside and noncontiguous to work hours	42 (11)		1 (2)		12 (5)		<.001	
	Contiguous to work hours	21 (11)		17 (7)		26 (13)		.03	
**Duration of inbox work on workdays and nonworkdays (min)**									
	Work hours inbox duration	25.36 (13.03)		47.97 (13.35)		42.13 (16.56)		.002	
	Outside work hours inbox duration	41.37 (13.81)		10.91 (5.63)		26.97 (13.26)		<.001	
	Inbox duration on nonworkdays	32.74 (37.46)		11.13 (19.69)		6.54 (11.3)		.03	
**Message types (percentage of all inbox time)**									
	Patients	32 (10)		35 (10)		42 (10)		.02	
	Results	30 (9)		32 (11)		26 (10)		.10	
	Requests	24 (7)		20 (6)		21 (6)		.31	
	Admin	14 (5)		13 (4)		11 (4)		.14	

Physicians in group 1 were more likely to batch their inbox work (ie, do most of their inbox work in a few chunks of time rather than consistently throughout the day) than group 2, as 50% (5/10) of physicians in group 1 batched their inbox work compared with 6% (1/17) in group 2 (*X*^2^_1_=4.03; *P*=.045). The rate of switching windows within the EHR was not statistically different among the 3 groups (*P*=.24), with all groups switching windows 4-4.5 times per minute of EHR use, on average. The groups spent different amounts of time per message (*P*=.004). The time per message was higher for group 1 (mean 0.46 min, SD 0.11 min) than for group 2 (mean 0.35 min, SD 0.06 min) and group 3 (mean 0.38 min, SD 0.07 min). Groups 2 and 3 did not differ significantly (*P*=.21). In terms of inbox message types, there were statistically significant differences among groups in patient-initiated messages (*P*=.02), with group 3 spending a higher average percentage of their inbox time on patient-initiated messages than group 1, and no differences for other group pairs (Table 1).

### Stress Patterns

**Figure 2:**
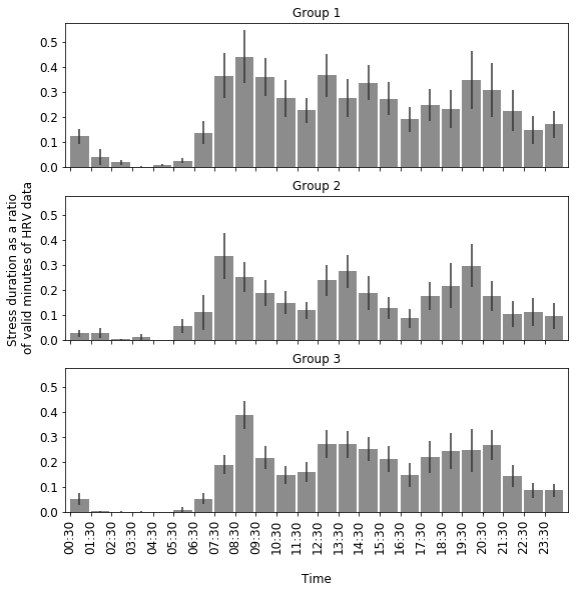
Workday stress patterns of each group. Error bars represent the SE of the mean. HRV: heart rate variability.

Visualizing stress patterns throughout the day showed that stress was high at the beginning of the workday. The first hour of work (8:30 AM to 9:30 AM) had an average stress duration of 35% of the hour (SD 26%; SE 4%). Stress then started to decrease until the lunch hour and increased again at the start of the afternoon clinic shift. Toward the end of the workday, the stress duration decreased. There was another increase in stress in the evening, followed by a decrease in stress at night and during typical sleep hours (Figure 2). This *3-wave* pattern of daily stress was consistent across the 3 work patterns, although group 2 had their highest stress an hour earlier (ie, 7:30 AM to 8:30 AM) than the other groups (Figure 2).

There was a difference in the average duration of stress during work hours among the groups (Kruskal-Wallis; *P*=.02). A posthoc comparison showed that group 1, the group with the highest after-hours inbox work duration, had a longer duration of stress during work hours than group 2 and group 3, with 33% (SD 27%) of work hours for group 1 being stressful (80 out of 243 min of valid HRV data indicated medium to high stress) compared with the 18% (SD 18%) for group 2 (47 out of 265 min of valid HRV data) and 22% (SD 24%) for group 3 (58 out of 265 min of valid HRV data). There was no significant difference between group 2 and 3 (*P*=.73). The number of valid minutes of HRV measurements was not significantly different across groups.

On average, physicians missed 45% (SD 20%; 9.4 out of 21) of the experience sampling prompts over the study period. Of the 485 submitted responses, 188 (38.8%) reported a stress level of over 50% (the midpoint of the slider). There was no significant difference in the average daily self-reported stress across the 3 inbox work patterns (*P*=.99).

Finally, in the survey on inbox management strategies and stress, physicians reported that 60% (SD 19%) of their work-related distress came from inbox management. Regarding the question of how distressful they find inbox management overall, of the 44 physicians, 19 (43%) said it was moderately stressful, 15 (34%) said it was very stressful, 6 (14%) said it was extremely stressful, and 4 (9%) said it was not very stressful. There were no statistically significant differences in survey responses across the 3 inbox work patterns.

### EHR Use Characteristics Associated With Stress

We investigated detailed EHR use characteristics associated with stress using a mixed effects model, with workdays as the unit of analysis. The model showed that fixed effects accounted for 15% of the variation in duration of stress during work hours (Table 2). The physician’s age, sex, and FTE worked were not associated with stress. The rate of switching windows when using the EHR was positively associated with stress (*P*=.001). Time spent on inbox work during work hours was positively associated with stress (*P*<.001), whereas time spent on other EHR activities during work hours was negatively (but very weakly) associated with stress (*P*<.001). Inbox work outside of work hours was positively associated with stress during work hours (*P*<.001). Interestingly, the proportion of inbox time spent on patient messages was not associated with stress. Surprisingly, batching inbox work for the day was also positively associated with stress (*P*<.001). Finally, days of the week were predictive of stress, with Mondays and Thursdays negatively associated with stress, whereas Tuesdays and Wednesdays positively associated with stress (*P*<.001 for each).

**Table 2:** Generalized linear mixed effects regression model.

Fixed effects^a^	β (SE)	Standard β^b^	*P* value
Full-time equivalent	1.94 (1.39)	.27	.16
Age	−.01 (.02)	−.05	.79
Female	.45 (.38)	.21	.24
Window switching rate	.1 (.03)	.08	.001
Work hours inbox duration	.003 (.001)	.08	<.001
Work hours noninbox EHR^c^ duration	−.002 (0)	−.06	<.001
Nonwork hours inbox duration proportion	.35 (.07)	.09	<.001
Patient messages proportion	−.09 (.08)	−.01	.28
Batching	.13 (.03)	.06	<.001
Monday	−.22 (.04)	−.10	<.001
Tuesday	.16 (.03)	.06	<.001
Wednesday	.53 (.03)	.20	<.001
Thursday	−.13 (.04)	−.05	<.001

^a^The dependent variable is duration of stress during work hours. Friday is the reference category for the variable *day of week*.

^b^Standard β is the standardized coefficient.

^c^EHR: electronic health record.

## Discussion

### Principal Findings

To our knowledge, this study is the first to measure physician stress using wearable sensors over several days of outpatient practice and the first to identify distinct EHR inbox work patterns and their associations with stress. Although the topic of EHR use and stress (specifically, self-reported burden, burnout, workload, and well-being) has been addressed in previous studies, this study is novel in that we measured stress unobtrusively and continuously through physiologic measures and used system logs to gain detailed insight about EHR use factors associated with stress. Higher rates of EHR window switching, longer inbox work duration, and a higher proportion of inbox work done outside of work hours were associated with higher stress. Daily stress patterns showed 3 waves of stress: in the first hour of work, at or after lunch hours, and in the evening.

In addition, we found that physicians fell into 3 groups with different patterns of inbox work. Some physicians tended to do most of their inbox work within work hours, whereas others did inbox work before or after but contiguous to work hours. The third group did inbox work in late evenings. These groups differed in characteristics such as inbox work batching, time per message, and the proportion of inbox time spent on patient messages. Physicians who did most of their inbox work outside of work hours were more likely to batch email and spend more time per message, whereas physicians who mostly do their inbox work within work hours were more likely to continually check their inbox throughout the workday, potentially in the short periods of time between patient appointments, and spent less time per message. The group that did most of their inbox work outside of work hours had the longest stress duration during work hours.

A strength of this study is that we measured stress using 3 different methods. The HRV-based stress provided a continuous timestamped stress measure that could be correlated with inbox use patterns throughout the day, the experience sampling measure provided momentary self-assessment of stress 3 times a day, and the survey provided a reflective measure on perceived overall stress related to inbox work. HRV-based stress differed across groups but self-report measures did not. It is well established in the literature that short-term self-reported (ie, perceived) stress and acute physiological stress do not always align linearly in daily life settings [48-50]; however, both are important to monitor as they both have health and well-being implications [51-54].

### Comparison With Previous Work

Previous studies on EHR use patterns have quantified the time spent on different EHR activities within and outside of work hours [14,24]. However, variation among physicians is not well studied, and no previous study has attempted to characterize physicians based on their patterns of daily inbox use. One study [16] found that physician-to-physician variation explains most of the variability in EHR use time. We extend the findings on the variation in EHR use, focusing on inbox use and comparing physician characteristics across work patterns based on work hours and after-hours EHR inbox use. Aligned with previous findings [16], we did not find differences in physicians’ sex distributions between the group with the longest after-hours inbox time and the group with the shortest after-hours inbox time. We also did not find differences based on FTE, contrary to previous findings [16] that more work relative value units generated by physicians (another measure of workload) were associated with more EHR time after work hours.

Most studies use basic measures to characterize EHR usage, such as the duration of time [14,15,55]. In one study, researchers used more complex measures to characterize mobile EHR usage, such as the number of log-ins and features used and usage paths (ie, the frequency and complexity of consecutive actions) [56]. They compared doctors across medical specialties and found that physicians other than surgeons had more diverse mobile EHR usage patterns with higher complexity and repetitive loops compared to surgeons [56]. In this study, we also used detailed EHR and inbox usage characteristics such as window switching, inbox work batching, the time per message, message types, and the time distribution between work and nonwork hours. Our finding that the window switching rate was positively associated with stress could reflect the complexity and repetitiveness of physicians’ EHR interactions, as indicated in prior work [56], and the efficiency issues often associated with physicians’ satisfaction with EHRs [57]. Another study on EHR inbox burden [8] also reported that excessive steps were needed to process messages and that physicians recommended reducing the number of mouse clicks necessary to process messages.

A recent study suggested a relationship between patient call messages and clinician burnout [58]. Their category of patient messages included all messages related to patient care tasks, such as phone calls, refill requests, and patient care forms. In our study, the category of patient messages included only patient-initiated messages and was not found to be associated with stress, although it comprised most of the inbox time for physicians.

It is not surprising that the differences among groups in HRV-based stress did not align with self-reported perceived stress. Previous studies have noted several issues in the interrelationship between perceived and physiological stress [59]. For example, the timing of the perceived stress prompt (before, during, or after a stressor event) could determine whether and how perceived stress correlates with physiological stress measured during the stressor event [60-62]. This has important implications for real-time stress monitoring for physicians, as it suggests that daily prompts to measure perceived stress in situ could fail to capture physiological stress. Increased and prolonged physiological stress reactions are associated with several health and well-being risks [63].

The results also suggest practical implications for organizational changes and system design. Previous studies have recommended a fundamental redesign of the EHR to improve data entry and retrieval [11]. On the basis of our finding that window switching is associated with stress, a redesign that minimizes the need to navigate to different windows to record or obtain information may be beneficial. For example, contextual information for inbox messages can be made visible from the inbox [8]. Our findings lend support to recommendations from a previous study to automate frequently performed actions such as message routing and leverage team support for inbox management [8]. Allocating time for inbox management within work hours, also recommended in a previous study, may also help reduce stress [8].

### Limitations

In this study, the regression model with EHR use characteristics explained 15% of the variation in duration of stress during work hours, which is a considerable proportion given the myriad factors that can potentially influence stress. However, stress was likely to have also been influenced by other variables that were beyond the scope of this study. In addition, the associations we observed between stress and window switching, inbox work duration, and inbox work outside work hours do not necessarily prove that the latter factors cause stress. It is possible that physicians who are busier during work hours have more stress and also make more window switches, have more inbox work, and have to do more inbox work outside work hours.

HRV-based measures are affected by several factors, such as health and physical activities. Although we tried to control these effects with our participant inclusion criteria and by removing periods that had physical activity registered by the wearable device, it is possible that carry-over effects of physical activity are still present in the HRV data of sedentary moments. Moreover, removing periods with physical activities could have removed periods when psychological stress was experienced. For example, walking to an important meeting could be mentally stressful but it will not be captured in our data because of the elimination of periods when walking is detected.

HRV data were excluded during periods of physical activities and were occasionally missing because of sensors losing contact with the skin. We set a minimum threshold (measurement period) of 20 minutes of valid data per hour for hourly stress measures and 2 hours for daily stress measures. Although not complete, we do feel that this is a reasonable proxy for the stress experience of that hour and day and a reasonable mitigation method for missing data.

Inbox use patterns might differ from one setting to another based on the organization’s policies and norms. For example, the medical group where this study was conducted encouraged patients to use EHR portal messages to communicate with physicians. Simultaneously, system-generated messages and administrative reminders are kept to a minimum whenever possible. Thus, the distribution of different message types may differ from that in other settings. These factors must be considered when generalizing our findings.

Finally, some physicians might have had panel management time (ie, time designated by departments specifically for tasks such as inbox management) incorporated within their work hours. In this study, we did not have access to data on panel management time. Thus, we cannot make assumptions about why inbox work patterns differed among physicians. We can only report the relationship of these different work patterns with stress.

### Conclusions

This study is the first to use continuous and unobtrusive measures of stress to evaluate associations between EHR inbox use and stress among physicians. A total of 3 potentially modifiable factors were associated with stress: window switching, inbox work duration, and inbox work outside work hours. These findings have implications for research and organizational policies on stress measurement and EHR inbox management time and EHR system design.

## Abbreviations

EDA: electrodermal activity
EHR: electronic health record
FTE: full-time equivalent
HRV: heart rate variability
PCP: primary care physician
TPMG: The Permanente Medical Group

## References

[1] Murphy DR, Meyer AN, Russo E, Sittig DF, Wei L, Singh H (2016). The burden of inbox notifications in commercial electronic health records. JAMA internal medicine.

[2] Renaud K, Ramsay J, Hair M (2006). "You've got e-mail!" ... shall I deal with it now? Electronic mail from the recipient's perspective. International Journal of Human-Computer Interaction.

[3] Barley SR, Meyerson DE, Grodal S (2011). E-mail as a source and symbol of stress. Organization Science.

[4] Mark G, Voida S, Cardello A (2012). "A pace not dictated by electrons": an empirical study of work without email. CHI '12: Proceedings of the SIGCHI Conference on Human Factors in Computing Systems.

[5] Tai-Seale M, Dillon EC, Yang Y, Nordgren R, Steinberg RL, Nauenberg T, Lee TC, Meehan A, Li J, Chan AS (2019). others. Physicians? Well-Being Linked To In-Basket Messages Generated By Algorithms In Electronic Health Records. Health Affairs ?.

[6] Lieu TA, Freed GL (2019). Unbounded?Parent-Physician Communication in the Era of Portal Messaging. JAMA pediatrics ?.

[7] Gregory Megan E, Russo Elise, Singh Hardeep (2017). Electronic Health Record Alert-Related Workload as a Predictor of Burnout in Primary Care Providers. Appl Clin Inform.

[8] Murphy Daniel R, Satterly Tyler, Giardina Traber D, Sittig Dean F, Singh Hardeep (2019). Practicing Clinicians' Recommendations to Reduce Burden from the Electronic Health Record Inbox: a Mixed-Methods Study. J Gen Intern Med.

[9] Gross James J, John Oliver P (2003). Individual differences in two emotion regulation processes: implications for affect, relationships, and well-being. J Pers Soc Psychol.

[10] Sato Hirotsune, Kawahara Jun-ichiro (2011). Selective bias in retrospective self-reports of negative mood states. Anxiety Stress Coping.

[11] Colicchio Tiago K, Cimino James J, Del Fiol Guilherme (2019). Unintended Consequences of Nationwide Electronic Health Record Adoption: Challenges and Opportunities in the Post-Meaningful Use Era. J Med Internet Res.

[12] Shanafelt TD, Dyrbye LN, Sinsky C, Hasan O, Satele D, Sloan J, West CP Relationship between clerical burden and characteristics of the electronic environment with physician burnout and professional satisfaction.

[13] Gardner R, Cooper E, Haskell J, Harris D, Poplau S, Kroth P, Linzer M (2019). Physician stress and burnout: the impact of health information technology. J Am Med Inform Assoc.

[14] Arndt BG, Beasley JW, Watkinson MD, Temte JL, Tuan W-J, Sinsky CA, Gilchrist VJ (2017). Tethered to the EHR: primary care physician workload assessment using EHR event log data and time-motion observations. The Annals of Family Medicine ?.

[15] Saag HS, Shah K, Jones SA, Testa PA, Horwitz LI (2019). Pajama Time: Working After Work in the Electronic Health Record. Journal of general internal medicine ?.

[16] Attipoe S, Huang Y, Schweikhart S, Rust S, Hoffman J, Lin S (2019). Factors Associated With Electronic Health Record Usage Among Primary Care Physicians After Hours: Retrospective Cohort Study. JMIR Hum Factors.

[17] Adler-Milstein J, Zhao W, Willard-Grace R, Knox M, Grumbach K (2020). Electronic health records and burnout: Time spent on the electronic health record after hours and message volume associated with exhaustion but not with cynicism among primary care clinicians. J Am Med Inform Assoc.

[18] Heponiemi T, Kujala S, Vainiomäki S, Vehko T, Lääveri T, Vänskä J, Ketola E, Puttonen S, Hyppönen H (2019). Usability Factors Associated With Physicians’ Distress and Information System–Related Stress: Cross-Sectional Survey. JMIR Med Inform.

[19] Vainiomäki S, Aalto A, Lääveri T, Sinervo T, Elovainio M, Mäntyselkä P, Hyppönen H (2017). Better Usability and Technical Stability Could Lead to Better Work-Related Well-Being among Physicians. Appl Clin Inform.

[20] Khairat S, Burke G, Archambault H, Schwartz T, Larson J, Ratwani R Focus Section on Health IT Usability: Perceived Burden of EHRs on Physicians at Different Stages of Their Career. Appl Clin Inform 2018 Apr;?.

[21] Palojoki Sari, Pajunen Tuuli, Saranto Kaija, Lehtonen Lasse (2016). Electronic Health Record-Related Safety Concerns: A Cross-Sectional Survey of Electronic Health Record Users. JMIR Med Inform.

[22] Chen Y, Ngo V, Harrison S, Duong V (2011). Unpacking Exam-Room Computing: Negotiating Computer-Use in Patient-Physician Interactions. CHI '11: Proceedings of the SIGCHI Conference on Human Factors in Computing Systems.

[23] Singh H, Spitzmueller C, Petersen NJ, Sawhney MK, Smith MW, Murphy DR, Espadas D, Laxmisan A, Sittig DF (2013). Primary care practitioners' views on test result management in EHR-enabled health systems: a national survey. J Am Med Inform Assoc.

[24] Akbar F, Mark G, Warton E, Reed M, Prausnitz S, East J, Moeller M, Lieu T (2020). Physicians' electronic inbox work patterns and factors associated with high inbox work duration. J Am Med Inform Assoc.

[25] Singh H, Spitzmueller C, Petersen NJ, Sawhney MK, Sittig DF (2013). Information overload and missed test results in electronic health record-based settings. JAMA Intern Med.

[26] Murphy Daniel R, Reis Brian, Sittig Dean F, Singh Hardeep (2012). Notifications received by primary care practitioners in electronic health records: a taxonomy and time analysis. Am J Med.

[27] Lieu Tracy A, Warton E Margaret, East Jeffrey A, Moeller Mark F, Prausnitz Stephanie, Ballesca Manuel, Mark Gloria, Akbar Fatema, Awsare Sameer, Chen Yi-Fen Irene, Reed Mary E (2021). Evaluation of Attention Switching and Duration of Electronic Inbox Work Among Primary Care Physicians. JAMA Netw Open.

[28] Ray Rebecca D, McRae Kateri, Ochsner Kevin N, Gross James J (2010). Cognitive reappraisal of negative affect: converging evidence from EMG and self-report. Emotion.

[29] Stone Arthur A., Turkkan Jaylan S., Bachrach Christine A., Jobe Jared B., Kurtzman Howard S., Cain Virginia S. (1999). The science of self-report: Implications for research and practice.

[30] Rajendra Acharya U, Paul Joseph K, Kannathal N, Lim CM, Suri JS (2006). Heart rate variability: a review. Med Biol Eng Comput.

[31] Malik M, Bigger JT, Camm AJ, Kleiger RE, Malliani A, Moss AJ, Schwartz PJ (1996). Heart rate variability: Standards of measurement, physiological interpretation, and clinical use. European Heart Journal.

[32] Stys A, Stys T (1998). Current clinical applications of heart rate variability. Clin Cardiol.

[33] Picard RW, Fedor S, Ayzenberg Y (2015). Multiple Arousal Theory and Daily-Life Electrodermal Activity Asymmetry. Emotion Review.

[34] Okkonen J, Heimonen T, Savolainen R, Turunen M (2018). Assessing Information Ergonomics in Work by Logging and Heart Rate Variability.

[35] Mark G, Iqbal S, Czerwinski M, Johns P, Sano A, Lutchyn Y (2016). Email Duration, Batching and Self-interruption: Patterns of Email Use on Productivity and Stress. CHI '16: Proceedings of the 2016 CHI Conference on Human Factors in Computing Systems.

[36] Koldijk S, Sappelli M, Neerincx M, Kraaij W (2013). Unobtrusive Monitoring of Knowledge Workers for Stress Self-regulation. Carberry S., Weibelzahl S., Micarelli A., Semeraro G. (eds) User Modeling, Adaptation, and Personalization. UMAP 2013. Lecture Notes in Computer Science.

[37] Lyu Y, Luo X, Zhou J, Yu C, Miao C, Wang T, Shi Y, Kameyama K (2015). Measuring Photoplethysmogram-Based Stress-Induced Vascular Response Index to Assess Cognitive Load and Stress. CHI '15: Proceedings of the 33rd Annual ACM Conference on Human Factors in Computing Systems.

[38] Mattingly S, Gregg J, Audia P, Bayraktaroglu A, Campbell A, Chawla N, Das SV, De CM, D'Mello S, Dey A, Gao G, Jagannath K, Jiang K, Lin S, Liu Q, Mark G, Martinez G, Masaba K, Mirjafari S, Moskal E, Mulukutla R, Nies K, Reddy M, Robles-Granda P, Saha K, Sirigiri A, Striegel A (2019). The Tesserae Project: Large-Scale, Longitudinal, In Situ, Multimodal Sensing of Information Workers. CHI'19 Extended Abstracts: Proceedings of CHI Conference on Human Factors in Computing Systems Extended Abstracts.

[39] Jessup G, Bian S, Chen Y-W, Bundy A (2012). PIEL Survey application.

[40] Stress and Recovery Analysis Method Based on 24-hour Heart Rate Variability Internet. Firstbeat Technologies Ltd.

[41] Fuller D, Colwell E, Low J, Orychock K, Tobin MA, Simango B, Buote R, Van Heerden D, Luan H, Cullen K, Slade L, Taylor NGA (2020). Reliability and Validity of Commercially Available Wearable Devices for Measuring Steps, Energy Expenditure, and Heart Rate: Systematic Review. JMIR Mhealth Uhealth.

[42] Gillinov S, Etiwy M, Wang R, Blackburn G, Phelan D, Gillinov A, Houghtaling P, Javadikasgari H, Desai M (2017). Variable Accuracy of Wearable Heart Rate Monitors during Aerobic Exercise. Med Sci Sports Exerc.

[43] Claes J, Buys R, Avila A, Finlay D, Kennedy A, Guldenring D, Budts W, Cornelissen V (2017). Validity of heart rate measurements by the Garmin Forerunner 225 at different walking intensities. J Med Eng Technol.

[44] Dooley Erin E, Golaszewski Natalie M, Bartholomew John B (2017). Estimating Accuracy at Exercise Intensities: A Comparative Study of Self-Monitoring Heart Rate and Physical Activity Wearable Devices. JMIR Mhealth Uhealth.

[45] Blascovich J, Tomaka J (1996). The biopsychosocial model of arousal regulation. Advances in experimental social psychology.

[46] Reynolds D (2015). Gaussian Mixture Models. Li S.Z., Jain A.K. (eds) Encyclopedia of Biometrics.

[47] Starczewski A, Krzyżak A (2015). Performance Evaluation of the Silhouette Index. Artificial Intelligence and Soft Computing. ICAISC 2015. Lecture Notes in Computer Science, vol 9120.

[48] Kageyama T, Nishikido N, Kobayashi T, Kurokawa Y, Kaneko T, Kabuto M (1998). Self-reported sleep quality, job stress, and daytime autonomic activities assessed in terms of short-term heart rate variability among male white-collar workers. Ind Health.

[49] Hernandez RJ (2015). Towards wearable stress measurement. PhD Dissertation.

[50] Muaremi A, Arnrich B, Tröster G (2013). Towards measuring stress with smartphones and wearable devices during workday and sleep. BioNanoScience ?.

[51] Rod N, Grønbaek M, Schnohr P, Prescott E, Kristensen T (2009). Perceived stress as a risk factor for changes in health behaviour and cardiac risk profile: a longitudinal study. J Intern Med.

[52] Lagraauw H Maxime, Kuiper Johan, Bot Ilze (2015). Acute and chronic psychological stress as risk factors for cardiovascular disease: Insights gained from epidemiological, clinical and experimental studies. Brain Behav Immun.

[53] VanItallie TB Stress: A risk factor for serious illness. Metabolism 2002 Jun;?.

[54] Vogelzangs Nicole, Beekman Aartjan T F, Milaneschi Yuri, Bandinelli Stefania, Ferrucci Luigi, Penninx Brenda W J H (2010). Urinary cortisol and six-year risk of all-cause and cardiovascular mortality. J Clin Endocrinol Metab.

[55] Anderson Jacob, Leubner Jason, Brown Steven R (2020). EHR Overtime: An Analysis of Time Spent After Hours by Family Physicians. Fam Med.

[56] Soh Ji Yeong, Jung Sang-Hyuk, Cha Won Chul, Kang Mira, Chang Dong Kyung, Jung Jaegon, Lee JeanHyoung, Choi Jong Soo, Kim Kyunga (2019). Variability in Doctors' Usage Paths of Mobile Electronic Health Records Across Specialties: Comprehensive Analysis of Log Data. JMIR Mhealth Uhealth.

[57] Williams Daniel Clay, Warren Robert W, Ebeling Myla, Andrews Annie L, Teufel Ii Ronald J (2019). Physician Use of Electronic Health Records: Survey Study Assessing Factors Associated With Provider Reported Satisfaction and Perceived Patient Impact. JMIR Med Inform.

[58] Hilliard R, Haskell J, Gardner R (2020). Are specific elements of electronic health record use associated with clinician burnout more than others?. J Am Med Inform Assoc.

[59] Hjortskov N, Garde AH, Ørbæk P, Hansen ?M (2004). Evaluation of salivary cortisol as a biomarker of self-reported mental stress in field studies. Stress and Health.

[60] Schlotz W, Kumsta R, Layes I, Entringer S, Jones A, Wüst Stefan (2008). Covariance between psychological and endocrine responses to pharmacological challenge and psychosocial stress: a question of timing. Psychosom Med.

[61] Gaab J, Rohleder N, Nater U, Ehlert U (2005). Psychological determinants of the cortisol stress response: the role of anticipatory cognitive appraisal. Psychoneuroendocrinology.

[62] Oldehinkel A, Ormel J, Bosch N, Bouma E, Van Roon Arie M, Rosmalen J, Riese H (2011). Stressed out? Associations between perceived and physiological stress responses in adolescents: the TRAILS study. Psychophysiology.

[63] Logan JG, Barksdale DJ (2008). Allostasis and allostatic load: expanding the discourse on stress and cardiovascular disease. J Clin Nurs.

